# Treatment of Scarlatina by Inunction

**Published:** 1850-03

**Authors:** 


### ARTICLE III.

*Treatment of Scarlatina by Inunction.*---By Dr. SCHNEEMAN. Translated by J. L. Milton, Esq.

[Dr. Schneeman, physician at the Hanoverian court, has published a work advocating a new mode of treating Scarlatina, which, he says, materially shortens the duration of the disease, and checks all infection at the end of the third or fourth day. Mr. Milton gives the following translation of those portions of the pamphlet which relate to treatment.]

From the first day of the illness, and as soon as we are certain as to its nature, the patient must be rubbed every morning and evening over the whole body with a piece of bacon, in such a manner that, with the exception of the face (?) and hairy scalp, a covering of fat is every where applied.--- In order to make this rubbing-in somewhat easier, it is best to take a piece of bacon the size of the hand, choosing a part still armed with the rind, that we may have a firmer grasp. On the soft side of this piece, slits are to be made in various directions, in order to allow the oozing out of the fat: and this is still further promoted by placing the bacon, for some time previously to using it, near the stove--in the oven, or on the hob. But it is not proper to make the friction with a warm piece, and the fat must be allowed to cool before using it.--- The rubbing must be most conscientiously performed, and, if considered necessary, the whole can be done under the bed-clothes. But such precaution as this is unnecessary; the children may safely get up. No injury will ever be done by it, and after once seeing it done, the mother or nurse soon learns to relieve the medical man of his task. Although this plan, from the mess it makes, is not calculated to find favor in the fine world---although it dirties bed and linen, as well as the persons of the children, yet the first few days of its application produce results which make all this forgotten, and inspire the mothers with enthusiasm for this method. With a rapidity bordering on magic, all, even the most painful symptoms of the disease are allayed; quiet, sleep, appetite, and good humor return, and there remains only the impatience to quit the sick room. To obtain this, however, other things are necessary besides mere infriction with fat, but still I think myself bound to impute the most important share of the merit to this peculiar treatment of the skin. The truth of this assertion will appear from a citation of the first results which follow on the treatment indicated.

1. The improbability, I might say impossibility, of the patient getting cold while the skin is thus covered with fat—a point in no disease more important than here; were this the sole benefit of the fatty infriction, it would still be of great service.

2. The dry brittleness of the skin, and the tormenting itching, are by it not only materially alleviated, but for the most part fully put a stop to. Every practitioner knows how often the itching and burning of the skin in scarlet fever are unendurable to children, keeping them constantly in distressing movement, and robbing them of sleep. Hence, children are generally well satisfied with this rubbing-in, and often ask for a repetition of the proceeding long before the time is come.

3. The influence on the physiological functions of the skin is still more important. During the coming-on of scarlet fever, the skin becomes diseased, in consequence of which it dies off. During this illness, and until a new covering is again prepared for the surface, the functions of the skin are naturally very faultily, or, during the desquamation, probably not at all, performed. In order to explain the extent and importance of the imperceptible functions of the skin, merely mechanically viewing the matter, I refer the reader to the accurate experiments of Seguin, which fix the quantity of matter thrown off from the outer skin at eleven grains per minute in a grown person, and thererefore more than two pounds per day. What efforts must it cost the organism to lead so large a quantity into other paths, in order to throw it off when the skin is incapable of doing so! But the preservation of the whole being is the first and most important law, and however great the effort may be, the other organs must perform this function.

[I must here observe, that at first Dr. Schneeman thought—and indeed' in this portion of his pamphlet, stated—that no desquamation takes place under this treatment, so that, in fact, the functions of the skin are maintained in full integrity. Later observation, however, convinced him that, with all possible care, desquamation to a certain extent will ensue. This he communicated to me, requesting me to insert it in the translation. To resume:]

4. With this disappearance of the desquamation disappear all those bad symptoms which attend on it. This feature alone of this method of treatment will have its weight with the practitioner in all ages. In order now to give a striking proof of the importance and the bad influence which the interrupted functions of the skin produce on the healthy activity of relative, even if distant organs, I may cite the fact, that death is always the result where more than one half of the skin has been destroyed by fire or boiling liquid. Sooner or later such is the case. A similar destruction of the skin ensues in scarlet fever, with this difference, that it takes place gradually, and thereby the organism is better enabled, by employing all the activity of the body, to find aid against the mischief which, to the injury of the patient, must result from the cessation of the functions of the skin.

5. The oxidation of the blood is thus considerably promoted, the interrupting of which is the cause of such serious phenomena. As the disease of the throat is not improbably in great part due to the interruption of the functions of the and lungs, it must naturally disappear, or not present itself, where these are continued in integrity; and such is found in practice to be the case.

6. Owing to the fatty covering, the skin is kept in a moist state, and the cuticle thrown off cannot be driven about the room by currents of air, as happens where the patient has been kept dry: and thus one fertile source of infection is kept closed up, it being well known that infection is most easily communicated at the period of desquamation. The author believes, not only that such is the case, but also that by this method the danger of infection under any circumstances is materially lessened with the disappearance of the eruption from the skin; inasmuch as the process of generating infectious material is interrupted by restoring the skin to its normal state, a point of which he need not point out the importance.

7. By shortening the period of desquamation, and protecting the patient against the sequela of the disease, the duration and treatment of this can be shortened to a period of six to ten days—an immense advantage to the patient and his relatives, particularly as he is always liable to relapses, and is, even in mild cases considerably weakened.

8. The remedy is (cheap), harmless, practical, and is perhaps never counter-indicated.

The linen must not be too frequently changed, as a clean shirt takes up more of the fatty matter than one already saturated, and hence the skin is sooner deprived of its fatty covering. The rubbing-in is to be kept up twice a day for three weeks, and once a day during the fourth. The patient is, after this, to be daily washed with cold water and soap, and then only is the warm bath to be commenced. This process does not, as has been argued in some colleges, interfere with the natural course of the malady, and thus expose the individual to second and third attacks. In severe cases, the remedy may be repeated three or four times within the twenty-four hours. The main point is to keep the skin *always* cool and moist; and here, even with all possible precaution, the skin will sometimes come off in certain places. But cases of this kind are only unimportant. The practitioner will do well to fix the exact hour at which the rubbing-in is to take place; it will then most probably be better and more regularly performed.—*London Lancet.*

